# Artificial Intelligence to Predict Major Arrhythmic Events Based on Left Ventricular Electroanatomic Mapping Data

**DOI:** 10.3390/jcm15083078

**Published:** 2026-04-17

**Authors:** Yari Valeri, Paolo Compagnucci, Marialucia Narducci, Paolo Veri, Emanuele Pecorari, Isabel Concetti, Giuliano Santagata, Giovanni Volpato, Francesca Campanelli, Leonardo D’Angelo, Martina Apicella, Vincenzo Schillaci, Giuseppe Sgarito, Sergio Conti, Roberto Scacciavillani, Francesco Solimene, Gemma Pelargonio, Antonio Dello Russo, Francesco Piva, Michela Casella

**Affiliations:** 1 Department of Biomedical Sciences and Public Health, Marche Polytechnic University, 60121 Ancona, Italy; 2 Cardiology and Arrhythmology Clinic, Marche University Hospital, 60126 Ancona, Italy; 3 Fondazione Policlinico Universitario A. Gemelli IRCCS, 00168 Rome, Italy; 4 Department of Information Engineering, Polytechnic University of Marche, 60121 Ancona, Italy; 5 Clinica Montevergine, 83013 Mercogliano, Italy; 6 Department of Internal Medicine, Division of Cardiology, Section of Cardiac Electrophysiology, University of Iowa, Iowa City, IA 52242, USA; 7 IRCCS Istituto Mediterraneo per i Trapianti e Terapie ad Alta Specializzazione, University of Pittsburgh Medical Center, 90133 Palermo, Italy; 8 Department of Cardiovascular and Thoracic Sciences, Catholic University of the Sacred Heart, Largo F Vito 1, 00168 Rome, Italy; 9 Department of Specialistic Clinical and Odontostomatological Sciences, Polytechnic University of Marche, 60121 Ancona, Italy; 10 Department of Clinical, Special, and Dental Sciences, Marche Polytechnic University, 60121 Ancona, Italy; 11 Maria Cecilia Hospital, GVM Care & Research, 48033 Cotignola, Italy

**Keywords:** artificial intelligence, ventricular arrhythmia, major arrhythmic events, left ventricle, electroanatomic mapping, machine learning, deep learning, support vector machine, linear regression

## Abstract

**Background/Objectives**: Electroanatomic mapping (EAM) provides high-resolution spatial and electrogram information, but the prognostic utility of quantitative EAM features has not been systematically evaluated with contemporary artificial intelligence (AI) methods. We investigated whether an AI analysis of quantitative EAM exports from the CARTO system enhances the prediction of major arrhythmic events (MAEs). **Methods**: In this retrospective, multicenter cohort study, 248 consecutive patients undergoing left ventricular EAM at four tertiary electrophysiology centers were analyzed. Numerical EAM descriptors (spatial coordinates, unipolar/bipolar voltages, local activation time, impedance) were transformed into derived metrics, including local activation heterogeneity (GR), late-potential extent (LAT), bipolar–unipolar discrepancy (VLT), and low-amplitude scar extent (Scar Areas), and were spatially normalized via spherical projection. Clinical, anamnestic, and imaging variables were integrated. Machine learning and deep learning models were trained with an 80:20 train/test split and evaluated using three-fold cross-validation. Performance metrics included area under the receiver operating characteristic curve (AUC), accuracy, sensitivity, specificity, and precision. **Results**: Models incorporating both clinical and AI-processed EAM features achieved high discriminatory performance (test AUC up to 0.92; accuracy up to 0.896). Specificity was consistently high (≈0.97–0.998), whereas sensitivity remained modest (≈0.39–0.58). Among the EAM-derived features, GR was the most consistently informative predictor across algorithms and analyses; VLT, LAT, and Scar Areas also contributed substantially. Regionally, basal sub-mitral, subaortic, and posterolateral basal-to-mid zones exhibited the strongest associations with MAEs. **Conclusions**: AI-driven quantitative analysis of left ventricular EAM exports augments risk stratification for MAEs beyond conventional clinical and binary EAM descriptors. Reflecting local conduction heterogeneity, GR emerged as the dominant EAM predictor. Prospective validation in larger, disease-specific cohorts and real-time integration within EAM platforms are warranted.

## 1. Introduction

Complex ventricular arrhythmias are among the leading causes of cardiovascular death and, in most cases, occur in patients with structural and/or genetic heart conditions involving the left ventricle [1]. Structural alterations of the left ventricle can be assessed using non-invasive imaging techniques, such as electrocardiography, echocardiography, and cardiac magnetic resonance imaging (MRI) [2]. Electroanatomic mapping (EAM) allows for the evaluation of endocardial and epicardial potentials within the ventricular chambers, enabling the identification of regions with reduced amplitude endocardial potentials, late potentials, or fragmented potentials. Several studies have already confirmed the diagnostic and prognostic role of EAM in predicting major arrhythmic events. These studies already demonstrate a good correlation between the EAM-derived parameters and arrhythmic recurrence, even though the analyses were based on relatively crude mapping parameters. Notably, this association appears to be independent of traditional clinical variables [3,4,5,6].

Artificial intelligence (AI) refers to a field of computer science that focuses on developing algorithms and computational models to enable computer systems to simulate human-like intelligent behavior and critical thinking [7,8].

In recent years, both medicine and cardiology have increasingly integrated AI, with notable applications in electrocardiography and non-invasive imaging techniques, such as MRI [9,10,11]. However, to the best of our knowledge, there are no studies in the literature addressing the application of AI in the study and interpretation of endocavitary electrical potentials obtained through EAM. The aim of this study is to assess the utility and performance of left ventricular EAM data extracted from the CARTO system and analyzed using artificial intelligence, as well as their correlation with MAEs. This study will assess whether and how these values are correlated with major arrhythmic events during follow-up.

## 2. Materials and Methods

### 2.1. Study Setting

We conducted a retrospective, observational, multicenter study to evaluate the utility of artificial intelligence in predicting major arrhythmic events (MAEs) through the analysis of numerical values extracted from the CARTO system software (Version 7) obtained from EAM of the left ventricle, alongside clinical, anamnestic, and imaging data. A total of 248 consecutive patients who underwent EAM of the left ventricle with the CARTO navigation system were enrolled from four tertiary-level electrophysiology centers in Italy: 220 patients from the University Hospital Ospedali Riuniti of Ancona, 6 patients from the Montevergine Clinic in Avellino, 18 patients from the Agostino Gemelli University Polyclinic, and 4 patients from the Ospedali Riuniti Villa Sofia Hospital in Palermo. Patients were referred to the centers for one of the following reasons: ablation of non-idiopathic ventricular ectopy, ablation of ventricular tachycardia, or EAM for arrhythmic risk stratification, which may include an endomyocardial biopsy. Patients meeting the inclusion and exclusion criteria were enrolled. The inclusion criteria included (1) age ≥ 18 years, (2) provision of informed consent to participate in the study and to undergo the EAM procedure, and (3) presence of structural heart disease requiring EAM of the left ventricle. The exclusion criteria included (1) inability to retrieve all the necessary clinical data, (2) inability to extract the CARTO EAM file, or (3) inability to perform an adequate follow-up. All pre-procedural clinical, anamnestic, laboratory, and imaging data were meticulously collected for each patient. The numerical values extracted from the CARTO software obtained from EAM were analyzed using AI to develop predictive models for MAEs. This study was conducted in full compliance with institutional standards, national legal requirements, and the Helsinki Declaration for ethical standards. All patients provided written informed consent for the procedure.

### 2.2. Electroanatomic Mapping of the Left Ventricle

**Figure 1:**
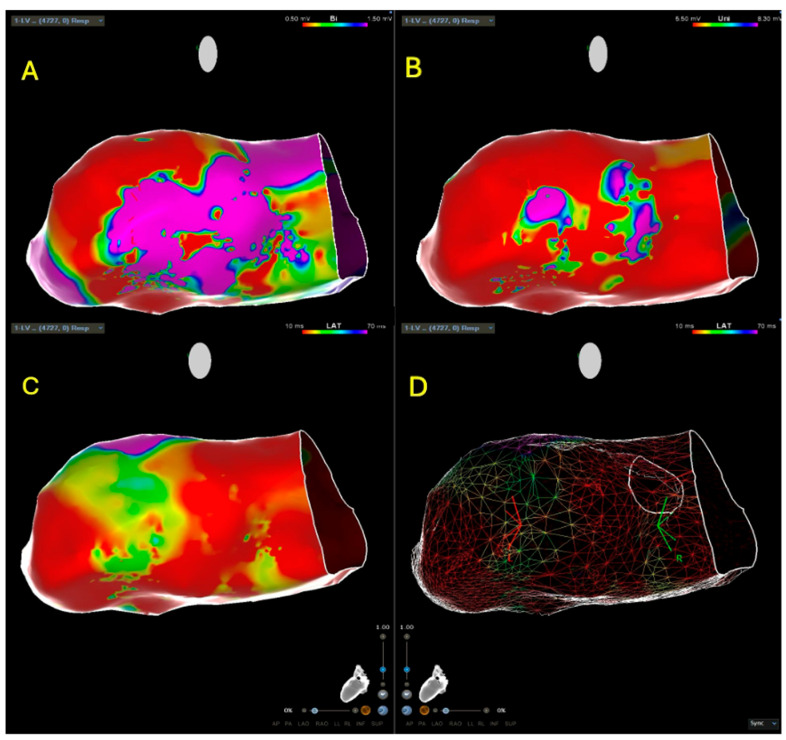
All images depict the posterior portion of the left ventricle. (**A**) shows a bipolar endocardial voltage map of the left ventricle. The color scale indicates healthy endocardial voltages in purple, borderline potentials within the so-called border zone in green, and low voltage pathological signals in red, corresponding to dense scar tissue. (**B**) shows the same left ventricular projection displaying unipolar signals, which reflect the epicardial regions of the ventricle. Purple indicates healthy tissue, whereas red identifies areas of dense scar. (**C**) illustrates a late potential substrate map, in which early healthy potentials are highlighted in red, while late potentials occurring after the surface QRS complex are shown in purple. (**D**) presents a MESH LAT image, allowing for more precise visualization of the different regions and projections of the left ventricle.

The procedure was performed under conscious sedation or general anesthesia. The electrophysiological procedure, including electroanatomic mapping of the right and/or left ventricle (endocardial and/or epicardial) and endomyocardial biopsy, has been extensively described in previous publications with regard to timing, methodology, and procedural technique [12,13,14,15]. The approach to the left ventricle, depending on the region of interest, was performed via the retroaortic, transseptal, or combined approach. In some cases, the outer limits of the ventricular chamber were already addressed using intracardiac echocardiographic reconstruction, which was then integrated with the EAM, using the CartoSound™ Module (Biosense Webster Inc., Irvine, CA, USA). Electroanatomic reconstructions of the left ventricular chamber were obtained using the CARTO™ 3 mapping system version 7 (Biosense Webster Inc., Irvine, CA, USA). Substrate mapping was performed in sinus rhythm with a contact force-sensing catheter (QDOT Micro™ or SmartTouch™ catheter; Biosense Webster Inc., Irvine, CA, USA). Only points with adequate catheter–tissue contact (>3 g) were acquired; for each point, the system automatically recorded the standard bipolar electrogram and the unipolar electrogram. Operators could also use a multipolar catheter with 5 or 8 arms, each equipped with 4 electrodes, each 1 mm in diameter with 2–6–2 mm inter-electrode spacing (PentaRay™ or OcataRay™; Biosense Webster, Inc., Irvine, CA, USA), recording bipolar electrograms between electrode pairs were spaced 2 mm apart (mini-bipolar electrograms). Conventional cutoffs were used to define regions of dense scar tissue (<0.5 mV) and border zones (0.5–1.5 mV) on the standard bipolar maps. Simultaneously, myocardial regions showing fragmented and late potentials were annotated (Figure 1).

### 2.3. Follow-Up

Monitoring for any MAE in patients who underwent left ventricular EAM and who had implantable devices (defibrillators, pacemakers, or loop recorders) was conducted through semi-annual in-office visits and, when available, remote monitoring. All patients were subjected to in-office cardiology visits at 3–6 months and 12 months post-procedure. Subsequent follow-up for patients not under the care of the center where the procedure was performed was conducted via telephone. MAEs were defined as sustained ventricular tachycardias or those treated with ATP/shock from the implantable devices, as well as ventricular fibrillations treated with ATP/shock.

### 2.4. Export and Extraction Algorithms

For this study, a computer equipped with an i9 processor and 64 GB of RAM was used. As for the software, MATLAB version R2023b was employed, both in its online and desktop versions. Additionally, the Parallel Computing Toolbox was implemented to enable parallel computing, maximizing the use of all processor cores. By executing calculations in parallel, the risk of idle cores is avoided, which significantly reduces computation time during program execution.

Following EAM with the CARTO mapping system, a file containing the results can be downloaded from the system. The export includes several pieces of information for each mapped point at the left ventricular endocardium. The most relevant extractable data include the following:

- Position Coordinates: Three coordinates for each of the three spatial axes (x, y, and z), which help determine the point’s position.
- Angular Coordinates: The three angles that define the catheter’s orientation relative to a fixed reference system during signal acquisition.
- Unipolar Voltage Value: Measures the electrical activity at a specific point in the heart using a single electrode, recording the amplitude of the electrical signal.
- Bipolar Voltage Value: Uses two electrodes placed at a certain distance from each other to record the electrical activity between them, capturing the signal amplitude.
- Local Activation Time (LAT): Indicates the precise moment when the heart cells at a specific point activate during the cardiac cycle. LAT represents the time elapsed from the start of the cardiac cycle or the electrocardiographic (ECG) wave until the heart cells’ activation in a specific region, helping to identify delays or advances in activation.
- Impedance Value: Expressed in ohms, it refers to the voltage difference between two points in the electrical circuit (e.g., between electrodes). Impedance, influenced by the resistance of the heart tissue, is used to assess the quality of the tissue being mapped.

From the exportable data from the EAM system, complex parameters were established to analyze using specific algorithms that the system does not have by default. To meet these needs, specific algorithms were created to calculate the following information:

- Point-to-point difference of bipolar voltage from unipolar voltage: the numerical value is obtained by subtracting the unipolar voltage value from the bipolar voltage value for each collected point. This analysis helps to assess the intramyocardial voltage and to identify regions where several points with high differences are clustered in the same area. Points with differences exceeding a certain threshold (the last 20% between the maximum and minimum values) are considered critical. Regions with clusters of critical points are identified, analyzed, and their extension is calculated as a percentage of the total points. This parameter is referred to in the study as “VLT”. A significant finding for this parameter is when high values are found clustered in a specific area.
- Deceleration areas or Gradient Value: these are zones where a very early potential and a very late potential are found within a short distance, indicating regions with a significant difference in activation times. The numerical value is obtained by subtracting the earliest activation time from the latest activation time and dividing the area by the distance between the two points. Studying these areas helps identify cardiac regions where there is a rapid deceleration of cardiac conduction, potentially highlighting critical isthmuses and regions involved in the genesis and maintenance of cardiac arrhythmias. This parameter is referred to as “GR”. A significant finding for this parameter is when high values are found clustered in a specific area.
- Late potential extension evaluation: this evaluates regions where the latest potentials are clustered. The LAT values are used for all projections. Values below a certain threshold (the last 20% between the maximum and minimum values) are considered late potentials. Adjacent late points are considered part of the same delay area, allowing for the extension and position of late potentials to be calculated as a percentage of non-late potentials. This parameter is referred to as “LAT”. A significant finding for this parameter is when high values are found clustered in a specific area.
- Low-amplitude potential extension evaluation: this evaluates regions where low-amplitude potentials, recorded as “scar”, are clustered. Values below a certain threshold (<0.5 mV) are considered low amplitude. This parameter is referred to as “Scar Areas”. A significant finding for this parameter is when low values are found clustered in a specific area.

Given that the heart of each patient has different sizes and spatial orientations, it was decided to normalize these values by projecting them onto a sphere. A fixed-size sphere is created, and its surface is divided into 14,400 equal-area faces. The centroid of the resulting point cloud from the measurements is calculated and positioned at the center of the sphere, with all points projected from the centroid onto the inner surface of the sphere. Each face of the sphere can either have a zero value if no projection hits that face, or the value of the projected point if it does. The number of faces was chosen so that no face contains more than one projected point, thereby avoiding approximations that would reduce the accuracy of the analysis. The main advantages of the sphere projection are spatial normalization—since the coordinates of the faces and the sphere’s dimensions are fixed, it is possible to compare mappings from different patients—and ease of interpretation, as the algorithm automatically extracts not only the point-by-point values, but complex parameters derived from interactions between the points.

**Figure 2:**
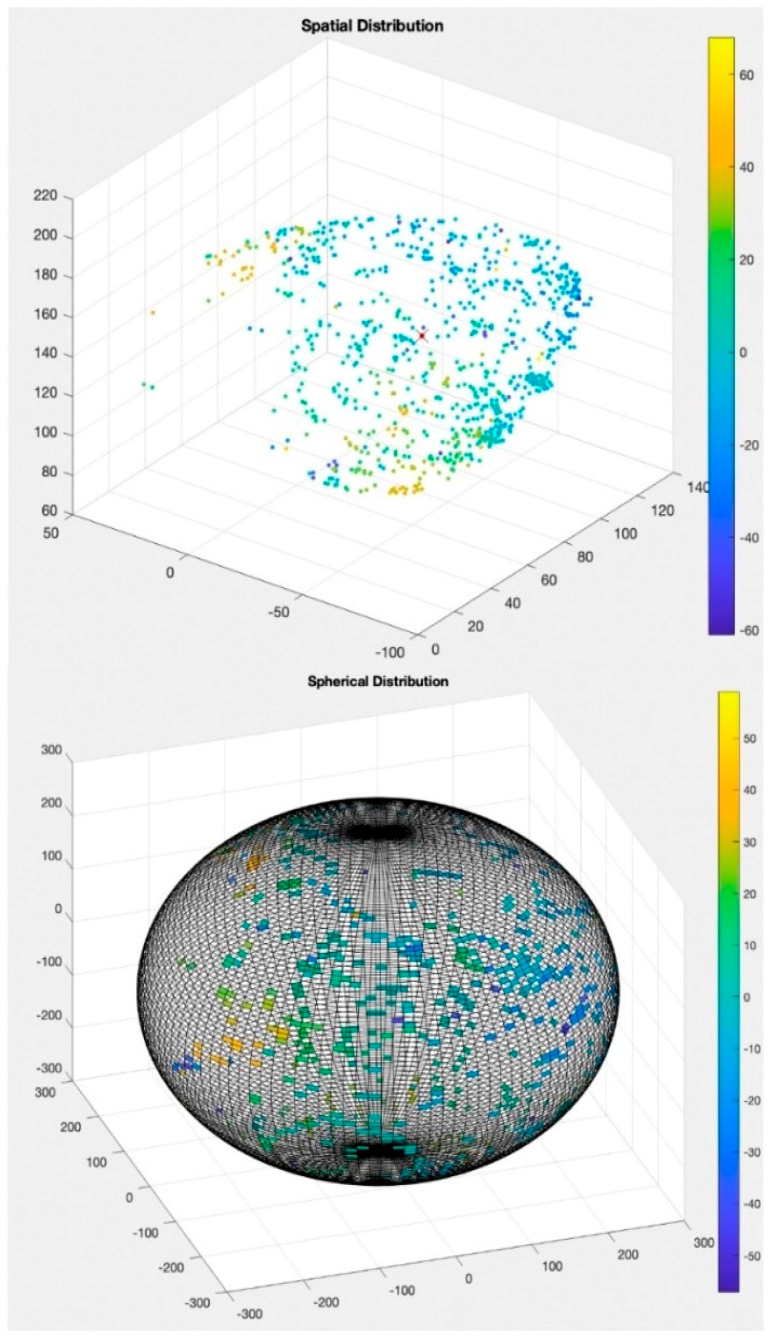
Spherical representation of the projection of the analyzed points.

**Figure 3:**
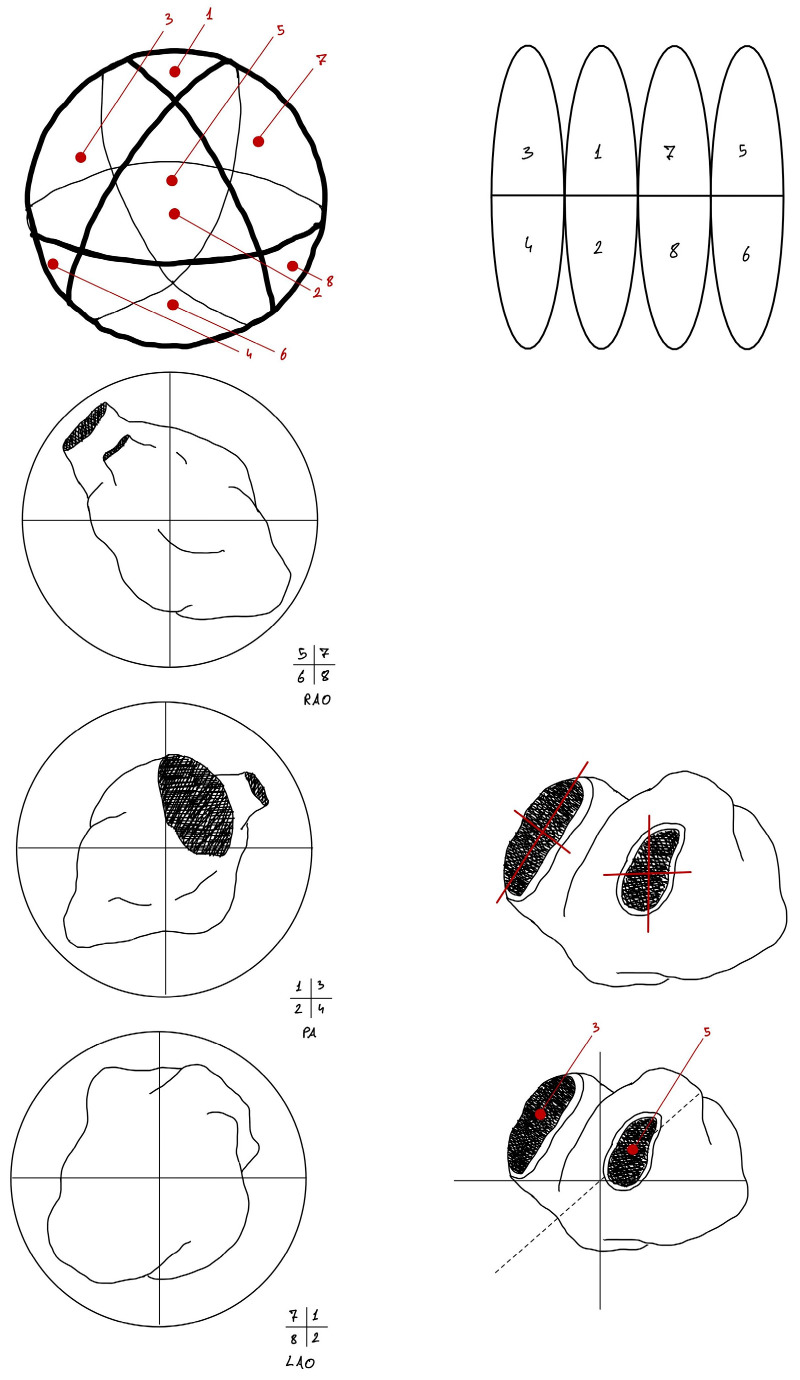
Subdivision of the heart into eight regions corresponding to specific projection points on the spherical surface. The cardiac regions have been divided into 8 different areas, which have been numbered using Roman numerals, each corresponding to a specific region. In particular: 1, anterior sub-mitral region; 2, posterior mid-apical region; 3, antero-septal mitro-aortic valvular region; 4, postero-septal mid-apical region; 5, antero-lateral sub-valvular mitro-aortic region; 6, postero-lateral mid-basal region; 7, antero-lateral mid-basal region; 8, apical and infero-apical mid-apical region.

For further analysis, the sphere upon which the points were projected was subdivided into 8 regions representing macro-cardiac projection regions. Each portion of the sphere corresponds to a specific cardiac region, allowing for the projection and assignment of any point in EAM to a precise cardiac region (Figure 2 and Figure 3).

In addition to the parameters derived from EAM, several clinical variables were also considered in the artificial intelligence analysis and in the development of the predictive models. The clinical parameters included in the analyses are listed in the Supplementary Materials.

### 2.5. Training, Validation and Testing

The dataset was split into training and testing sets, with 80% allocated for training and 20% for testing. Given the limited number of available samples, the K-fold cross-validation technique was chosen to assess the model. The test set data were not used for model training, simulating real-world scenarios where the model encounters previously unknown variables. Cross-validation is a technique that extends this approach by repeatedly splitting the data into distinct training and testing sets (referred to as “folds”) with different combinations. This iterative process can be repeated as many times as necessary to obtain an average evaluation of the model’s performance. By repeating this process, the risk of overfitting the model to the data is reduced, enhancing the model’s ability to make generalizable predictions for a broader population. In this study, three folds were used. The performance of the various models was evaluated using the following parameters: area under the curve (AUC), sensitivity, specificity, accuracy, and precision.

### 2.6. Study Endpoint

The primary endpoint of this study is to evaluate the utility and performance of EAM data analysis through AI and its correlation with MAEs. Another key primary endpoint within this analysis is to assess which of the studied parameters has the highest performance in predicting MACEs (major adverse cardiac events). The secondary endpoint of this study is to evaluate the advantage offered by the AI-based analysis of EAM compared to the exclusive analysis of clinical and anamnestic parameters in predicting MAEs.

## 3. Results

The results obtained include the performance values of the various models for each of the machine learning techniques employed. The results presented below represent parameter combinations that allow for, within a certain degree of accuracy, the identification of whether a patient may exhibit an MAE. These are not the only possible parameter combinations; however, for each analysis, the combination with the highest performance was selected.

A preliminary remark is that linear relationships yield explicit formulas with interpretable coefficients, thus allowing real weighting of each model parameter. By contrast, nonlinear relationships behave as “black boxes”: the data are processed and outputs are generated, but the internal computational steps used by the algorithm to obtain those results remain unknown.

We considered the sum of missing values. Specifically, any variable with less than 10% non-missing values was excluded. We assessed the heterogeneity within each variable. Variables with the same value across all patients were excluded, as they did not contribute meaningful information to the analysis. Variables with at least 5% of values differing among patients were retained. Variables dependent on subjective clinical operator choices—such as the type of mapping system or the choice of mapping/ablation catheter—were excluded, as they do not influence the underlying pathology.

The baseline characteristics of the patients, including clinical and anamnestic parameters, as well as echocardiographic, electrocardiographic, and laboratory data, are described in the Supplementary Materials.

### 3.1. Analysis of Parameters Independently of Cardiac Regionality

The six best-performing models obtained with each of the previously described algorithms were reported and analyzed. Table 1 lists the variables assigned to each model and enables the identification of the most frequently occurring parameters, allowing for the assignment of frequency of occurrence within the linear regression analysis.

The performance values associated with these models are reported in Table 2.

From Table 3, a comparative analysis of all the variables listed above can be derived.

Table 4 presents the variables assigned to each model and allows for the identification of the most frequently occurring parameters, thereby assigning them a frequency according to the linear SVM analysis.

The performance values associated with these models are reported in Table 5.

The performance values associated with these models are reported in Table 6.

Table 7 presents the variables assigned to each model and allows for the identification of the most frequently occurring parameters, thereby assigning them a frequency according to the nonlinear SVM analysis.

The performance values associated with these models are reported in Table 8.

**Figure 4:**
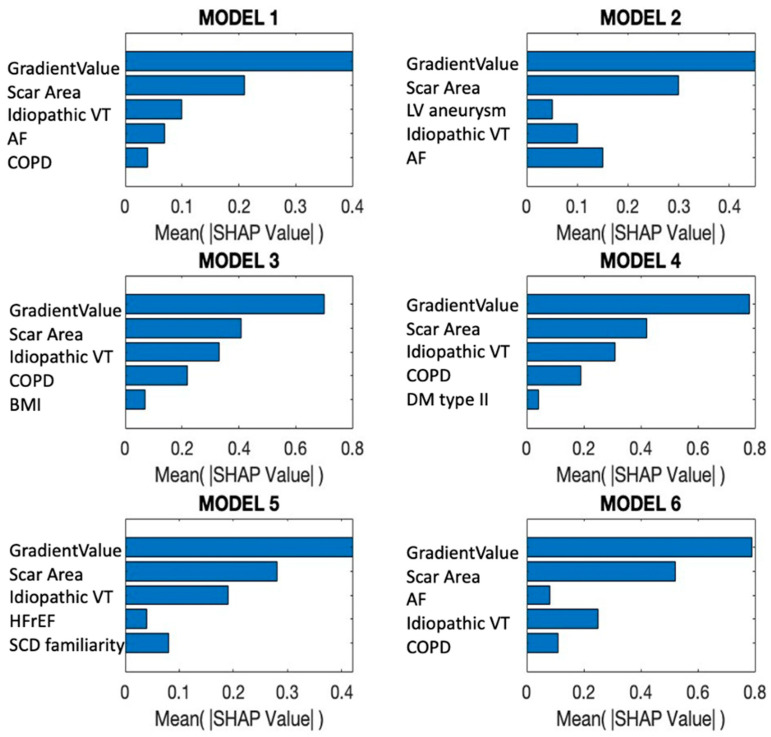
SHAP values for the features of each nonlinear support vector machine model with an RBF kernel. AF: atrial fibrillation; BMI: body mass index; COPD: chronic obstructive pulmonary disease; DM: diabetes mellitus; HFrEF: heart failure reduced ejection fraction; LV: left ventricle; SCD: sudden cardiac death; VT: ventricular tachycardia.

To understand the actual contribution of each variable to the output, as interpreted through SHAP values, Figure 4 is particularly informative.

Considering the analysis using deep learning and neural networks, Table 9 presents the variables assigned to each model, allowing for the identification of the most frequently occurring parameters and their corresponding frequency.

The performance values associated with these models are reported in Table 10.

### 3.2. Analysis of Parameters in Relation to Cardiac Regionality: CARTO Parameter Analysis

Independently of the clinical values, data extracted from the CARTO EAM software were evaluated. Table 11 presents the performance data for analyses conducted using linear regression and SVM.

The variables shown are those of the greatest importance, exhibiting the strongest correlation with MAEs (Table 12).

### 3.3. Analysis of Parameters in Relation to Cardiac Regionality: Analysis of Clinical Parameters Integrated with CARTO Parameters

Subsequently, data obtained from EAM were integrated with the patients’ clinical and anamnestic data and analyzed using both machine learning and deep learning approaches. Table 13 reports the performance values for the various analytical methodologies.

Table 14 present the variables with the highest predictive coefficients for MAEs, analyzed using linear regression (LR) and SVM.

Table 15 present the variables with the highest predictive coefficients for MAEs, obtained from nonlinear analyses with coefficient estimation using SHAP values.

## 4. Discussion

### 4.1. Main Findings

To the best of our knowledge, this is the first study to apply AI to EAM exports for the prediction of MAEs. From the data extractable from EAM exports, we analyzed the following four parameters: the gradient value (GR), representing the local transition from late to early potentials over short distances; the extent of late potentials (LAT); the difference between bipolar and unipolar potentials (VLT); and the extent of low-amplitude potentials.

Previous studies analyzing EAM data for the prediction of complex ventricular arrhythmic events are limited and generally evaluate substantially smaller EAM datasets compared with those analyzed in the present study. A bipolar-to-unipolar myocardial voltage ratio greater than 0.58, as well as the presence of extensive Scar Areas and widespread late potentials, have previously been correlated with an increased risk of recurrence after ablation [3,4,5,6].

Key results from the AI analysis of the EAM data are as follows:

- Compared with models based on medical history, clinical and instrumental data, or simple dichotomous EAM markers (presence/absence of low/late potentials), an AI analysis of EAM exports yields more powerful predictive models by exploiting quantities of information that traditional statistical methods cannot effectively handle.
- EAM-derived features are frequently included among the best predictive variables for MAEs and show strong associations with outcomes.
- A regional analysis reveals that certain EAM-derived parameters correlate strongly with MAEs in specific cardiac regions.
- When combined with clinical variables, EAM-derived AI features generally dominate the predictive models; notably, GR consistently demonstrated high predictive value.

### 4.2. MAE Prediction Using AI—Models Independent of Cardiac Regionality

We initially constructed predictive models for MAEs using both clinical variables and EAM-derived features. AI methods evaluated many possible models and selected those with the highest AUC and accuracy. The best-performing algorithms were linear regression (LR), SVM with linear and nonlinear kernels, and ANN. Across methods, EAM-derived parameters were the most frequently represented variables in high-performing models; GR was present in every top model and emerged as the single EAM parameter most strongly associated with MAEs. In nonlinear SVM models, variable importance measures identified GR as the dominant predictor. The parameter LAT and the Scar Areas frequently recur in the best-performing models, and in the variable-importance analysis they emerge, after the GR value, as the most influential predictors in the models. Among clinical variables, New York Heart Association (NYHA) class, systolic pulmonary arterial pressure (PAP), history of arrhythmic storm, hearth failure with reduced ejection fraction, and tricuspid annular plane systolic excursion (TAPSE) were relevant predictors, consistent with the prior literature on ventricular arrhythmic risk [16,17,18,19,20]. Model performance was high by several metrics (AUC > 0.9, accuracy > 0.8, specificity > 0.9), while sensitivity was modest (≈0.5–0.6), likely reflecting the large number of candidate features relative to the modest cohort size.

Simple binary EAM descriptors (presence/absence of low or late potentials) were not selected by AI, whereas continuous or AI-processed EAM features contributed consistently. This suggests that conventional EAM outputs are insufficient for comprehensive MAE prediction and that AI can extract additional prognostically relevant information.

### 4.3. MAE Prediction Using AI—Regional Analysis

We next analyzed clinical and AI-processed EAM data after assigning mapping points to anatomical regions. AUC, accuracy, and specificity remained high, while sensitivity again remained limited for the reasons noted above. A regional analysis made it possible to evaluate the EAM-derived parameters within specific cardiac areas. Both machine learning (LR, SVM) and deep learning (ANN) approaches showed that the EAM-derived metrics outperform clinical, historical, and imaging variables for MAE prediction. We identified regions with particularly strong predictive values; clinical predictors such as TAPSE, indexed left ventricular end-diastolic volume (iLVEDV), PAP, LVEF, and history of arrhythmia remained important [16,17,18,19,20,21,22].

Among the EAM-derived variables, GR again demonstrated the highest predictive performance across models. IMP and VLT contributed to a lesser extent. The regions most associated with predictive performance were zone 3, zone 1, and zone 5 (basal sub-mitral, subaortic, and basal-to-apical posterolateral areas). GR—computed as the LAT difference between nearby points—reflects local electrical heterogeneity. LAT (the overall extent of late potentials) was less predictive, which suggests that MAE generation may depend more on local heterogeneity and conduction block than on the total area affected.

Although prior EAM studies have not reported this specific correlation, cardiac MRI studies have shown that late-gadolinium enhancement (LGE) entropy and heterogeneity predict MAEs in hypertrophic, non-compaction, and ischemic cardiomyopathies, which supports our findings [21,22,23,24,25]. AI applications to MRI have already been used to predict arrhythmic circuits and potential VT substrates, reinforcing the trend toward personalized, image-guided risk stratification. Integration of EAM and non-invasive imaging may further optimize disease management [24,26,27,28].

Despite high-resolution mapping catheters rapidly providing extensive information, frequent arrhythmic recurrences after ablation imply that important targets may remain unrecognized. Traditional EAM identifies low-voltage areas, scars, and late potentials, features often found in critical tachycardia isthmuses whose ablation improves prognosis. An AI-based EAM analysis may extract additional parameters that better correlate with prognosis and help identify novel ablation targets [29,30,31].

### 4.4. Future Perspectives

Our study highlights GR as a highly predictive EAM-derived parameter for MAEs, yet current EAM systems do not calculate or display GR in real time. Embedding an AI algorithm into EAM software to generate a “heterogeneity map” could enable the identification of high-risk regions and an evaluation of whether targeted ablation of these areas reduces MAE incidence on follow-up.

### 4.5. Study Limitations

The main limitations of this study are as follows.

Heterogeneous indications for left ventricular EAM: patients underwent mapping for varied reasons (non-idiopathic PVC ablation, VT ablation, or risk stratification).

Heterogeneous underlying cardiac disease, including ischemic, hypertrophic, arrhythmogenic, non-ischemic dilated cardiomyopathy, myocarditis, and arrhythmic valve prolapse.

Relatively small sample size compared with a large number of variables, which limits generalizability and likely contributed to low sensitivity; a substantially larger cohort is required for robust, generalizable predictive modelling.

## 5. Conclusions

Left ventricular EAM remains a central diagnostic and therapeutic tool, particularly when combined with catheter ablation, but its full prognostic potential is underutilized. Novel EAM-derived parameters that are not routinely assessed can be strong predictors of MAEs when analyzed with AI. The GR value was the most consistently predictive parameter, outperforming classical EAM descriptors and many clinical predictors. Alterations in basal sub-mitral, subaortic, and posterolateral mid-basal regions were particularly associated with MAEs when abnormal EAM-derived parameters were present.

## Figures and Tables

**Table 1:** Models obtained through linear regression.

Variables	Model 1	Model 2	Model 3	Model 4	Model 5	Model 6
NYHA class	X	X	X	X	X	X
sPAP	X	X	X	X	X	X
LAT	X		X		X	
GR	X	X	X	X	X	X
HFrEF				X		
Hypertension	X					
Male Sex		X				X
Arrhythmic storm		X	X	X	X	X

GR: gradient value; HFrEF: heart failure reduced ejection fraction; LAT: local activation time; NYHA: New York heart association; sPAP: systolic pulmonary artery pressure.

**Table 2:** Performance values of the models obtained through linear regression.

	AUC Train	AUC Test	Accuracy	Precision	Sensibility	Specificity
MODEL 1	0.997	0.975	0.881	0.700	0.537	0.960
MODEL 2	0.980	0.934	0.875	0.696	0.532	0.951
MODEL 3	0.991	0.925	0.867	0.691	0.529	0.948
MODEL 4	0.986	0.925	0.848	0.687	0.527	0.945
MODEL 5	0.986	0.925	0.837	0.685	0.527	0.941
MODEL 6	0.986	0.925	0.833	0.685	0.525	0.940

**Table 3:** Coefficients of the polynomial resulting from the linear logistic regression analyses.

	MODEL 1	MODEL 2	MODEL 3	MODEL 4	MODEL 5	MODEL 6
Intercept	−21.007	−22.114	−12.052	−26.637	−12.457	−21.434
NYHA Class	3.889	5.161	1.281	5.060	2.697	2.548
PAP	0.262	0.279	0.144	0.313	0.124	0.383
LAT	0.034		0.163		0.043	
GR	0.492	0.682	0.375	0.441	0.271	0.223
HFrEF				2.104		
Hypertension	2.638					
Sex		98.661				104.115
Arrhythmic storm		2.808	2.808	0.208	1.223	3.089

GR: gradient value; HFrEF: heart failure reduced ejection fraction; LAT: local activation time; NYHA: New York heart association; PAP: systolic pulmonary artery pressure.

**Table 4:** Most frequently occurring variables among the six models selected using linear support vector machine.

VARIABLES	MODEL 1	MODEL 2	MODEL 3	MODEL 4	MODEL 5	MODEL 6
EF	X			X		
TAPSE	X	X	X			
PAP	X	X	X	X	X	X
Arrhythmic storm	X	X	X	X	X	X
**GR**	X	X	X	X	X	X
T-wave inversion			X	X		
Creatinine					X	
**VLT**					X	
**LAT**		X				X

EF: ejection fraction; GR: gradient value; LAT: local activation time; PAP: systolic pulmonary artery pressure; TAPSE: tricuspid annular plane systolic excursion; VLT: point-to-point difference of bipolar voltage from unipolar voltage.

**Table 5:** Coefficients of the polynomials resulting from the linear support vector machine analyses.

	MODEL 1	MODEL 2	MODEL 3	MODEL 4	MODEL 5	MODEL 6
Intercept	−8.301	−3.474	−1.096	−2.207	−9.353	−1.096
EF	0.046			0.936		
TAPSE	0.119	0.04	0.076			
PAP	1.963	0.234	0.287	0.179	0.297	0.335
Arrhythmic storm	1.963	3.635	4.217	4.906	4.751	3.971
GR	0.155	0.148	0.338	0.175	0.288	0.111
T-wave inversion			2.573	1.769		
Creatinine					1.268	
VLT					0.328	2.408
LAT		0.064				0.007

EF: ejection fraction; GR: gradient value; LAT: local activation time; PAP: systolic pulmonary artery pressure; TAPSE: tricuspid annular plane systolic excursion; VLT: point-to-point difference of bipolar voltage from unipolar voltage.

**Table 6:** Performance values of the analysis conducted using linear support vector machine.

	AUC Train	AUC Test	Accuracy	Precision	Sensibility	Specificity
MODEL 1	0.996	0.992	0.884	0.708	0.538	0.961
MODEL 2	0.993	0.988	0.871	0.701	0.536	0.956
MODEL 3	0.993	0.988	0.867	0.692	0.535	0.952
MODEL 4	0.984	0.979	0.851	0.655	0.532	0.949
MODEL 5	0.984	0.979	0.821	0.648	0.529	0.942
MODEL 6	0.984	0.979	0.817	0.619	0.528	0.940

**Table 7:** Most frequently occurring variables among the models obtained using nonlinear support vector machine.

VARIABLES	MODEL 1	MODEL 2	MODEL 3	MODEL 4	MODEL 5	MODEL 6
Scar Areas	X	X	X	X	X	X
GR	X	X	X	X	X	X
Paroxysmal VT	X	X	X	X	X	X
LV aneurysm		X				
AF	X	X				X
COPD	X		X	X		X
HFrEF					X	
BMI			X			
Diabetes mellitus				X		
Family history of SCD					X	

AF: atrial fibrillation; BMI: body mass index; COPD: chronic obstructive pulmonary disease; GR: gradient value; HFrEF: heart failure reduced ejection fraction; LV: left ventricular; SCD: sudden cardiac death; VT: ventricular tachycardia.

**Table 8:** Performance values of the analysis conducted using nonlinear support vector machine.

	AUC Train	AUC Test	Accuracy	Precision	Sensibility	Specificity
MODEL 1	0.995	0.986	0.889	0.724	0.537	0.969
MODEL 2	0.994	0.984	0.878	0.711	0.535	0.961
MODEL 3	0.986	0.967	0.867	0.692	0.534	0.953
MODEL 4	0.983	0.967	0.854	0.675	0.533	0.949
MODEL 5	0.967	0.945	0.832	0.644	0.530	0.947
MODEL 6	0.954	0.945	0.822	0.629	0.528	0.944

**Table 9:** Most frequently represented variables in the artificial neural network analysis.

VARIABLES	MODEL 1	MODEL 2	MODEL 3	MODEL 4	MODEL 5	MODEL 6
Male Sex	X	X	X	X	X	
Diabetes mellitus	X			X		
NYHA Class	X	X	X		X	X
Paroxysmal VT	X	X	X	X		
GR	X	X	X	X	X	X
COPD		X				
AF HFrEF			X	X		
PAP					X	X
BMI						X
TAPSE						X

AF: atrial fibrillation; BMI: body mass index; COPD: chronic obstructive pulmonary disease; GR: gradient value; HFrEF: heart failure reduced ejection fraction; NYHA: New York heart association; PAP: systolic pulmonary artery pressure; TAPSE: tricuspid annular plane systolic excursion; VT: ventricular tachycardia.

**Table 10:** Performance values obtained using artificial neural networks.

	AUC Train	AUC Test	Accuracy	Precision	Sensibility	Specificity
MODEL 1	0.993	0.986	0.887	0.710	0.537	0.965
MODEL 2	0.990	0.982	0.874	0.702	0.535	0.963
MODEL 3	0.986	0.978	0.861	0.686	0.533	0.956
MODEL 4	0.986	0.978	0.843	0.662	0.531	0.952
MODEL 5	0.984	0.974	0.817	0.631	0.530	0.950
MODEL 6	0.984	0.973	0.812	0.612	0.529	0.948

**Table 11:** Performance values for logistic linear regression (LLR) and support vector machine (SVM) analyses.

	AUC Train	AUC Test	Accuracy	Precision	Sensibility	Specificity
LLR	0.805	0.830	0.770	0.588	0.344	0.623
SVM	0.803	0.828	0.768	0.528	0.374	0.602

**Table 12:** Coefficients of the polynomials resulting from the linear regression analyses and support vector machine analyses.

PREDICTOR	COEF	PREDICTOR	COEF
‘(INTERCEPT’)	0.637	‘(INTERCEPT’)	1.0520
GR1	0.1140	GR1	0.8100
GR3	0.1350	GR3	0.8070
GR7	0.2220	GR7	0.7800
VLT1	1.5870	VLT1	1.0060
VLT4	10.5800	VLT3	0.6795
VLT3	0.8820	VLT5	0.3390
VLT8	4.1950	VLT7	0.4310
IMP3	0.5470	IMP3	0.5080
IMP5	0.3930	IMP4	0.5670
VLT6	1.9020	IMP8	0.6470

**Table 13:** Performance values for logistic linear regression (LLR), support vector machine (SVM), kernel-based support vector machine, and artificial neural network (ANN) analyses.

	AUC Train	AUC Test	Accuracy	Precision	Sensibility	Specificity
LLR	0.850	0.887	0.786	0.588	0.390	0.970
SVM	0.828	0.892	0.825	0.600	0.453	0.990
Kernel	0.890	0.920	0.855	0.680	0.583	0.998
ANN	0.875	0.890	0.896	0.696	0.500	0.998

**Table 14:** Coefficients of the polynomials resulting from the linear regression analyses and support vector machine analyses.

PREDICTOR (LR)	COEF	PREDICTOR (SVM)	COEF
‘(INTERCEPT’)	0.3783	‘(INTERCEPT’)	0.6514
iLVEDV	1.7983	Arrhythmic Storm	0.8792
GR1	10.1258	iLVEDV	0.7426
GR4	6.3616	Valvular Card.	0.5383
TAPSE	1.5344	VLT3	0.6795
GR7	5.4059	IMP3	0.5662
GR3	4.1610	GR1	0.3592
IMP4	0.6218	HFpEF	0.4796
IMP3	0.2342	GR3	0.4716

iLVEDV: indexed left ventricular end diastolic volume; HFpEF: heart failure preserved ejection fraction; TAPSE: tricuspid annular plane systolic excursion.

**Table 15:** Coefficients of the polynomials resulting from the kernel SVM analyses and from the ANN analyses.

PREDICTOR	COEF	PREDICTOR	COEF
Arrhythmic Storm	1.721	Male Sex	137
LVEF	0.965	Arrhythmic Storm	1.880
TAPSE	0.633	Prior Stroke/TIA	1.880
PAP	1.324	iLVEDV	1.880
GR5	0.736	GR3	0.6420
GR7	0.984	GR5	0.6420
GR1	1.332	VLT5	0.6420
VLT3	2.448	VLT6	0.6420
VLT5	0.347	IMP6	0.6420
IMP3	1.421		

iLVEDV: indexed left ventricular end diastolic volume; LVEF: left ventricular ejection fraction; PAP: systolic pulmonary artery pressure; TAPSE: tricuspid annular plane systolic excursion; TIA: transient ischemic attack.

## Data Availability

The data presented in this study are available upon request from the corresponding author due to privacy and legal reason.

## Supplementary Materials

The following supporting information can be downloaded at: https://www.mdpi.com/article/10.3390/jcm15083078/s1, Table S1: The table summarizes the clinical and anamnestic characteristics, as well as the underlying structural heart disease, of the overall study population, the subgroup without arrhythmic recurrence, and the subgroup with arrhythmic recurrence; Table S2: The table summarizes the echocardiographic, electrocardiographic, laboratory, and EAM characteristics of the overall study population, the subgroup without arrhythmic recurrence, and the subgroup with arrhythmic recurrence.

## Abbreviations

The following abbreviations are used in this manuscript:

ACT	Activated clotting time
AI	Artificial intelligence
AUC	Area under the curve
DL	Deep learning
EAM	Electroanatomic mapping
LR	Linear regression
MACEs	Major adverse cardiac events
MAEs	Major arrhythmic events
ML	Machine learning
MMH	Maximum-margin hyperplane
NYHA	New York heart association
SVM	Support vector machine
PAP	Systolic pulmonary arterial pressure
RBF	Radial basis function
ReLu	Rectified linear unit
VT	Ventricular tachycardia
