# Supplementary material for: Artificial Intelligence to Predict Major Arrhythmic Events Based on Left Ventricular Electroanatomic Mapping Data
Source: J Clin Med. 2026 Apr 17;15(8):3078. doi: 10.3390/jcm15083078 (PMC13116828; doi:10.3390/jcm15083078)
Supplement: Supplementary file 1 [file jcm-15-03078-s001.zip › jcm-4173495-supplementary.pdf]

Supplementary materials

The clinical parameters included in the analyses are listed above:

- Age
- Sex
- BMI, body mass index
- Hypertension
- Diabetes mellitus
- Chronic kidney disease
- Smoke
- Sudden cardiac death familiarity
- Hyperthyroidism
- COPD, chronic obstructive pulmonary disease
- OSAS, obstructive sleep apnoea syndrome
- Prior stroke ore transitory ischemic attack
- Coronary disease (prior by-pass or revascularization)
- Hearth failure: preserved, mild-reduced, reduced ejection fraction
- NYHA class
- Prior syncope
- Type of cardiopathy (dilatative, arrhythmogenic, ischemic, hypertrophic, myocarditis, amyloidosis, sarcoidosis)
- Channelopathies
- Left ventricular ejection fraction
- Indexed left ventricular end diastolic volume
- TAPSE, Tricuspid Annular Plane Systolic Excursion
- Mitral regurgitation
- Pulmonary arterial pressure
- Left ventricular aneurysm
- T-wave inversion
- Low voltage
- Haemoglobin
- Creatinine
- Arrhythmic storm
- Paroxysmal VT
- Post-procedural therapy: Amiodarone, Beta-blocker, Flecainide, Angiotensin Receptor-Neprilysin Inhibitor, Angiotensin Receptor Blockers, ACE inhibitor, Mineralocorticoid Receptor Antagonists.
- Low voltage area
- Scars area
- Late potential
- Follow-up duration (month)

	ALL PATIENTS (N=248)	NO ARRHYTHMICS EVENTS (N=198)	ARRHYTHMICS EVENTS (N=50)	P VALUE
**AGE (YEARS) - MEDIAN [IQR]**	62 [48,7-74]	60 [45,3-72]	69 [55,5-77]	**P= 0,005**
**MALE SEX - N (%)**	193 (78)	149 (75)	44 (88)	P= 0,053
**BMI - MEDIAN [IQR]**	26 [24-28]	26 [24-28]	27 [25-29]	**P= 0,009**
**HYPERTENSION - N (%)**	112 (45)	81 (41)	31 (62)	**P= 0,007**
**DIABETES MELLITUS – N (%)**	24 (10)	16 (8)	8 (16)	P= 0,091
**EGFR < 60 ML/MIN/M² – N (%)**	39 (16)	25 (13)	14 (28)	**P= 0,008**
**SMOKE – N (%)**	80 (32)	57 (29)	23 (46)	**P= 0,012**
**SCD FAMILIAIRTY– N (%)**	20 (8)	15 (8)	5 (10)	P= 0,574
**ATRIAL FIBRILLATION – N (%)**	44 (18)	27 (14)	17 (34)	**P<0,001**
**HYPERTHYROIDISM – N (%)**	8 (3)	6 (3)	2 (4)	P= 0,729
**COPD – N (%)**	14 (6)	9 (5)	5 (10)	P= 0,135
**OSAS – N (%)**	15 (6)	11 (6)	4 (8)	P= 0,517
**PRIORE STROKE – N (%)**	16 (6)	10 (5)	6 (12)	P= 0,074
**PRYOR SYNCOPE – N (%)**	24 (10)	16 (8)	8 (16)	P= 0,090
**HEARTH FAILURE**				
**PRESERVED EF – N (%)**	5 (2)	4 (2)	1 (2)	P= 0,993
**MILD REDUCE EF – N (%)**	39 (16)	32 (16)	7 (14)	P= 0,707
**REDUCE EF – N (%)**	85 (34)	55 (28)	30 (60)	**P<0,001**
**NYHA CLASS - MEDIAN [IQR]**	1 [1-2]	1 [1-2]	2 [1 - 2,75]	**P<0,001**
**II – N (%)**	57 (23)	37 (19)	20 (40)	**P= 0,001**
**III – N (%)**	29 (12)	16 (8)	13 (26)	**P<0,001**
**IV – N (%)**	1 (0)	1 (0)	0 (0)	P= 0,615
**- ARRHYTHMIC STORM - N (%)**	59 (24)	38 (19)	21 (42)	**P< 0,001**
**- PAROXYSMAL TV - N (%)**	80 (32)	60 (30)	20 (40)	P= 0,190
**CARDIOMYOPATHY**				
**-DILATED– N (%)**	**37** (15)	**25** (13)	11 (22)	P= **0,093**
**-ISCHEMIC– N (%)**	**63** (25)	42 (21)	**21** (42)	P= 0,003
**-ARRHYTHMOGENIC– N (%)**	**8** (3)	**7** (4)	**1** (2)	P= 0,583
**-HYPERTROPHIC - N (%)**	**7** (3)	**6** (3)	**1** (2)	P= 0,694
**-VALVULAR - N (%)**	**33 (13)**	**27 (14)**	**6 (12)**	**P= 0,072**
**-AMYLOIDOSIS - N (%)**	4 (2)	1 (1)	3 (6)	P= 0,006
**-MYOCARDITIS - N (%)**	21 (8)	**18** (9)	**3** (6)	P= 0,483
**- CHANNELOPATHIES - N (%)**	1 (0)	1 (1)	0 (0)	P= 0,615

Table S1: The table summarizes the clinical and anamnestic characteristics, as well as the underlying structural heart disease, of the overall study population, the subgroup without arrhythmic recurrence, and the subgroup with arrhythmic recurrence.

	ALL PATIENTS (N=248)	NO ARRHYTHMICS EVENTS (N=198)	ARRHYTHMICS EVENTS (N=50)	P VALUE
**LVEF % – MEDIAN [IQR]**	52 [35-60]	55 [40-60]	35 [27 – 52,3]	**P< 0,001**
**ILVEDV (ML/M^2) - MEDIAN [IQR]**	69 [56-81,5]	66 [54-80]	75,5 [70-90,4]	**P< 0,001**
**TAPSE - MEDIAN [IQR]**	22 [19-25]	22 [19-25]	21 [19-23]	P= 0,315
**SEVERE MI - N (%)**	7 (3)	5 (3)	2 (4)	P= 0,573
**LV ANEURYSM - N (%)**	24 (10)	16 (8)	8 (16)	P= 0,091
**PAPS - MEDIAN [IQR]**	30 [25-35]	30 [25-35]	34,5 [28-38]	**P= 0,026**
**T WAVE INVERSION - N (%)**	47 (19)	42 (21)	5 (10)	P= 0,071
**ECG LOW VOLTAGE - N (%)**	10 (4)	9 (5)	1 (2)	P= 0,414
**HAEMOGLOBIN- MEDIAN [IQR]**	14 [13,1-14,9]	14,1 [13,1-15]	13,7 [12,9-14,55]	P= 0,076
**CREATININE - MEDIAN [IQR]**	0,96 [0,83-1,18]	0,94 [0,8-1,13]	1,03 [0,92-1,35]	**P= 0,007**
**POST-PROCEDURAL THERAPY**				
**-BETA-BLOCKER – N (%)**	178 (72)	136 (69)	42 (84)	**P= 0,032**
**-IC AAD – N (%)**	10 (4)	9 (5)	1 (2)	P= 0,414
**-AMIODARONE – N (%)**	77 (31)	51 (26)	26 (51)	**P< 0,001**
**-ARB/ACEI/SACUBRITIL-VALSARTAN- N (%)**	146 (58)	111 (56)	35 (70)	P= 0,074
**-MRA- N (%)**	77 (31)	54 (27)	23 (46)	**P= 0,011**
**LOW VOLTAGE AREAS - N (%)**	136 (55)	99 (50)	37 (74)	**P= 0,002**
**SCARS AREA - N (%)**	117 (47)	81 (41)	36 (72)	**P< 0,001**
**LATE POTENTIAL - N (%)**	110 (44)	71 (36)	39 (78)	**P< 0,001**
**FOLLOW-UP (MONTH) - MEDIAN [IQR]**	21 [12-35,2]	19 [11-33,5]	32 [14-40]	
**RECURRENCE MONTH - MEDIAN [IQR]**			6 [2-13]	

Table S2: The table summarizes the echocardiographic, electrocardiographic, laboratory, and EAM characteristics of the overall study population, the subgroup without arrhythmic recurrence, and the subgroup with arrhythmic recurrence.
